# Thoracic Ultrasonography in Calves: A Narrative Review of Techniques and Reporting Practices

**DOI:** 10.1111/jvim.70251

**Published:** 2025-09-30

**Authors:** George Lindley, John Donlon, Sébastien Buczinski

**Affiliations:** ^1^ Royal Veterinary College, Department of Pathobiology and Population Science Hertfordshire UK; ^2^ Department of Veterinary Medicine, Faculty of Science and Health Atlantic Technological University Letterkenny Co. Donegal Ireland; ^3^ Faculté de Médecine Vétérinaire Université de Montréal Quebec Canada

**Keywords:** cattle, pneumonia, thoracic ultrasonography, youngstock

## Abstract

Ultrasonography of the bovine lung is a noninvasive technique allowing recognition of lower respiratory tract lesions and differentiation from disease limited to the upper respiratory tract. Techniques for scanning the thorax have evolved to facilitate examination of cohorts of calves quickly, while maintaining accuracy. Classification systems for the interpretation of images, their assignment as normal or abnormal, and grading of their severity are varied. Without a reporting consensus, comparison of short‐and long‐term outcomes attributable to ultrasonographic findings is challenging. Differences in operator agreement might complicate interpretation further. The objective of this review was to gather methods for screening calf lungs using thoracic ultrasonography and describe the heterogeneity in scanning techniques and methods of image interpretation, including available scoring methods.

**Abbreviations**
BRD: bovine respiratory disease
ICS: intercostal space
qTUS: University of Ghent quick thoracic ultrasonography
TUS: thoracic ultrasonography

## 1 Introduction

Bovine respiratory disease (BRD) is a hypernym encompassing a complex of diseases with variable etiology, pathology, presentations, and case definitions. Antemortem diagnosis of BRD can be based upon behavioral changes, clinical signs, or using laboratory diagnostics, including the measurement of biomarkers, or via imaging techniques [1, 2]. Thoracic ultrasonography (TUS) is one method to improve the precision of BRD case management. It allows distinction between cases of lower and upper respiratory tract disease and allocation of antimicrobial therapy only to calves with clinical or subclinical pneumonia that are unlikely to self‐cure [3]. Objective measurement of lesions and characterization of severity is possible, with good correlation between TUS, radiography, and computed tomography [4]. Prognosis, as well as short‐ and long‐term implications can be inferred [5]. Strategically timed or serial measurements provide information regarding the temporality of disease within a herd, whilst treatment responses in individuals can also be assessed [6]. The duration of time taken to perform TUS examinations has been suggested as a potential factor precluding its use in clinical practice [7]. On this basis, examination methods focusing on specific regions of the thorax have been proposed, and simplified cut‐offs for defining treatment‐worthy animals suggested [8, 9, 10]. However, an increasing sum of TUS scanning methods and techniques for image interpretation complicates the assimilation of measured outcomes and the external validity of different available studies. The latter will also be affected by the reliability of the TUS operator, defined as their accuracy and precision to correctly identify positive and negative cases of disease [11, 12, 13]. This will vary by scanning method and method of image interpretation and will be affected by operator factors (intra‐operator agreement) as well as inter‐operator agreement, the agreement between different operators [14, 15]. A consistent approach to thoracic ultrasound in calves and the reporting of results might be helpful for the synthesis of research outcomes during future systematic reviews and meta‐analyses. The purpose of this narrative review based on an extensive literature search was to describe presently used techniques for TUS in the bovine calf and explore heterogeneity within published literature in order to suggest how future consensus between researchers and clinicians regarding the collection and reporting of TUS data might be achieved.

## 2 Materials and Methods

The objective of this review is: (i) to collate published methods for screening calf lungs using thoracic ultrasonography; (ii) to describe heterogeneity in ultrasonographic technique, image interpretation, operator reliability, and reported TUS outcomes; and (iii) to explain the limitations with TUS and its reporting as a first step toward reaching consensus in the reporting of studies on this topic.

To achieve the study objectives, a two‐step approach was undertaken. First, a literature search was conducted to identify primary research relevant to the topics of TUS technique, image interpretation, operator reliability and reported TUS outcomes. In the second step, these articles were considered within a broader context to inform narrative discussion, incorporating evidence from primary and secondary research including human studies.

### 2.1 Literature Search

A search strategy was developed according to the PRISMA 2020 checklist to identify primary studies using thoracic ultrasonography in calves that described novel ultrasonographic techniques or methods of image interpretation, outcome measures, or details of operator agreement. Two electronic databases, Pubmed and Web of Science, were searched for potentially relevant articles on 9th June 2025 using the keywords ((calf OR calves OR youngstock OR “young stock” OR “young bovines” OR cattle/OR bovine/) AND (“thoracic ultrasound” OR “thoracic ultrasonography” OR “thorax ultrasound” OR “thorax ultrasonography” OR “lung ultrasound” OR “lung ultrasonography” OR “respiratory ultrasound” OR “respiratory ultrasonography” OR “lung scanning” OR “thoracic scanning” OR “thorax scanning”) NOT human). An additional non‐structured search was also performed to identify oral abstracts and conference proceedings, inclusive of those available on the International Veterinary Information Service (IVIS). Since a search engine was not available for these databases, they were manually read.

### 2.2 Eligibility Criteria

In all cases, to identify articles eligible for inclusion, a single reviewer (GL) read the title and abstract of each record returned using the search terms and answered the questions: (a) does the title or abstract describe a research study or review article on the subject of thoracic ultrasound in calves? and (b) does the title or abstract describe the topics of thoracic ultrasound technique, image interpretation, operator reliability, or TUS outcomes? In a second step, the bibliographies of included articles were screened by one author (G.L.) to identify other relevant articles for inclusion. From this, a preliminary list of articles was generated and assessed for eligibility by all three reviewers (G.L., S.B., and J.D.) independently. During this process, articles were categorized if they presented novel data regarding TUS technique, image interpretation method, production outcome data, or inter/intra‐operator agreement. Production outcome data was defined as outcomes with long‐term effects on productivity, and articles presenting acute outcomes (e.g., hematological or behavioral changes and responses to treatment) were considered beyond the scope of this article and therefore were not eligible for inclusion. For any articles whose inclusion did not have unanimous agreement, consensus was reached after discussion between the authors.

All study designs, including calves of any origin (beef, dairy, or veal), were eligible for inclusion. Although keywords were used to search for papers on calves, no specific restriction on the age or weight of the study population was specified. The search was restricted to articles published after January 1st, 2000. Publications were only selected if they clearly stated the ultrasonographic technique and method of image interpretation that was performed and were written in the English language. To avoid redundancy, literature clearly duplicating previously published scanning techniques or interpretation methods was not considered unless it fulfilled other aspects of the study objectives. This was inclusive of review papers and book chapters. For each manuscript, specific data including the publication type, the number of calves enrolled, the number of TUS examinations performed, calf age, breed, sex, production system, location, image interpretation method, reported production outcomes, operator factors, and any reported data regarding inter‐ and intra‐operator agreement were recorded.

## 3 Results

### 3.1 Descriptive Statistics

**FIGURE 1:**
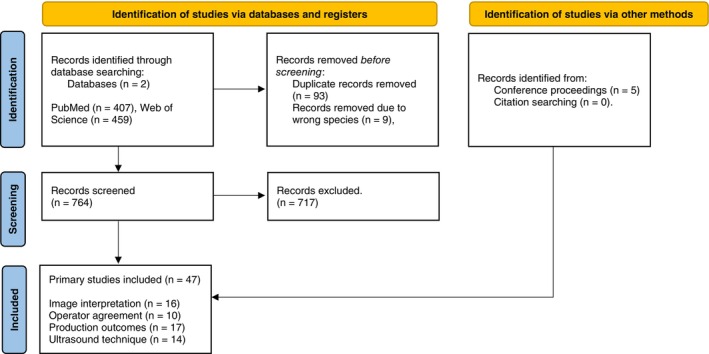
A PRISMA flowchart describing the literature search and study collection process.

A total of 407 and 459 publications were found using PubMed and Web of Science, respectively. Before screening, 93 duplicate articles were removed as well as 9 articles which were not related to cattle. During the screening process, 717 articles were excluded based upon the eligibility criteria. Information regarding study and TUS characteristics for retained articles (*n* = 47) was recorded into Microsoft Excel (Microsoft Office; Microsoft, Redmond, WA, USA) (Figure 1). The main descriptive characteristics of each article eligible for inclusion and the criteria they fulfilled for inclusion are described in Table S1. Risk of bias was not assessed.

In addition to papers retrieved through the search strategy, supplementary articles not specific to the aforementioned search were included if they contributed to the broader interpretive and critical aspects of the narrative review (*n* = 60). These additional sources were selected for their relevance to the discussion, for example, research on bovine lung development [16].

### 3.2 Scanning Techniques

#### 3.2.1 The Lung Fields

The lung fields of the bovine calf can be defined either by their adjacent structures on ultrasonography or their anatomical location when viewed externally. Dorsally, the lung fields begin ventral to the transverse vertebral processes. Depicted externally, the lung fields extend from the 10th to the 2nd intercostal space on the left and from the 10th to the 1st on the right [17]. The diaphragm separates thoracic and abdominal compartments and curves as it extends from a dorsal position at approximately the 10th rib space in a cranioventral direction until it terminates at around the level of the elbow, although its exact location varies during breathing [18]. The border of thorax and abdomen can be identified as an image containing both lung (seen dorsally) and spleen/liver (seen ventrally, on the left and right hand sides, respectively). The heart can be located at approximately the level of the elbow, whilst the thymus can be identified as a homogenous, hypoechoic structure separated by a hyperechoic mid‐septa located cranial to the heart but caudal to the internal thoracic artery and vein on the left hand side [19]. Externally, the cranial border of the lungs can be viewed as the cranial border of the thorax, or approximately dorsal and cranial to the elbow to reach the first intercostal space (ICS) on the right and the second ICS on the left [20]. A recognizable feature at the cranial boundary is the internal thoracic artery and vein. In larger animals, a thoracic inlet approach to reach these cranial regions is described, although uncommonly reported [21]. Finally, the ventral aspects of the lung fields vary by location, proceeding in a cranial direction from diaphragm caudally, past the costochondral junction, pleural deviation, and heart base.

The anatomic location of individual lung lobes viewed using TUS based upon ICS has also been described [17]. The left lung includes a cranial lobe, divided into a cranial (2nd–3rd ICS) and caudal part (4th–5th ICS), and a caudal lobe (6th–10th ICS). On the right side, the cranial lobe is divided once again into cranial (1st–2nd ICS) and caudal (3rd–4th ICS) parts, the former independently ventilated by a bronchus departing the trachea before the bifurcation [22]. Middle (5th ICS), caudal (6th–10th ICS) and accessory lobes are also present, the latter cannot be visualized using TUS. Knowledge of the ICS operators are imaging is essential when identifying specific lobes since they cannot be discriminated from lung images alone, although other anatomical landmarks (e.g., the heart) might be helpful. Simplified schemes which subdivide the thorax into cranial and caudal portions are suggested [9, 23].

### 3.3 Practical Techniques

Multiple techniques to perform TUS are described. When performing any technique, it is crucial to ensure the probe is oriented parallel to the surrounding ribs within each intercostal space. When positioned obliquely, the adjacent ribs cause acoustic shadowing and restrict the field of vision. A number of techniques involve systematic evaluation of all viewable lung fields [15, 24]. These incorporate either multiple passes of the probe from a caudal to cranial direction at between three and five different longitudes. Alternatively, starting at the most caudal ICS, proceed from dorsal to ventral, before progressing to the next ICS until the entire accessible lung has been imaged [17, 25].

More focused assessment techniques are also described. The UGhent quick TUS (qTUS) technique has been proposed as a method facilitating examination of all lung lobes with a single movement of the probe from caudal to cranial bilaterally. Starting from the caudodorsal thorax, the diaphragmatic lobes are visualized as the probe is passed in a cranioventral direction towards the elbow. Once a “half‐heart, half‐lung image” including the heart base is visualized, the probe is then passed cranially, dorsal then cranial to the heart base until the top of the cranial lobe is seen on both sides [26]. A more dorsal scan can also be performed in larger calves.

Other authors have examined specific regions of the thorax exclusively. Theoretically, by only considering the regions most commonly associated with disease, examination speed can be increased whilst minimizing loss of diagnostic sensitivity [7, 27]. This might explain why a focus has been paid to the cranial lobes [27, 28] or cranial and middle lobes [8, 27], specific intercostal spaces bilaterally [29, 30] or unilaterally [30, 31]. Examination of the most cranial ICS can be difficult for novice operators and is impractical in chute handling settings [32]. This might be why some authors avoid examination of the most cranial ICS [5, 33]. Others have limited exams to the more accessible diaphragmatic lobes bilaterally [34, 35] or unilaterally [36, 37], although these methods will be associated with a reduced sensitivity to detect lung consolidation [7]. Whilst more focused examination techniques can be advantageous in terms of examination time, other drawbacks will exist. Grading of severity in animals with widespread consolidation might be missed if consolidation is not identified in unconsidered regions. This could both affect the accuracy of prognostic assessments and impair the assessment of treatment success.

### 3.4 Image Interpretation

#### 3.4.1 A “Medical” Approach

The interpretation of pleural and lung ultrasonography is mostly based on what was initially developed from the human medical field and further adapted to common findings in calves [38]. We have classified approaches that define all specific (patho)physiological changes without using any scoring method as “medical” approaches. Reviewing all TUS anomalies is beyond the scope of this article, but herein we describe the basic normal and abnormal findings based upon human and veterinary literature.

It is important to keep in mind that the normal appearance of the lung is associated with the presence of a highly reflective sliding line which is composed of the visceral and parietal pleura sliding during breathing movements. The alveolar space is physiologically full of air and normal parenchyma cannot be seen due to the reverberation of the ultrasound beam at the visceral to alveoli interface [39]. Reverberation artifacts can also be seen which are manifested by the repetition of the same image attenuated “below” the pleural line. These horizontal lines are generally named as A‐lines and denote a normal lung ultrasound appearance. Several changes can affect the pleura, the pleural space (pleural effusion or pleural irregularity), or the pleura‐lung interstitial space and might be associated with the presence of vertical artifacts (various kinds of artifacts leading to “comet‐tail” or B‐line artifacts or coalescent comet‐tail‐like artifacts). The presence of occasional B‐line artifacts can be observed normally in many species of healthy animal [21, 40]. In human medicine, a threshold of ≥ 3 B‐line artifacts within a single intercostal space has been suggested as a criterion to define an interstitial pattern, although consensus is less clear regarding a similar threshold in calves [41]. Similarly, in humans an increased number of comet‐tails has been observed in interstitial lung diseases, wet lung syndromes with pulmonary edema as well as with viral pneumonia or as an early indicator of pre‐consolidation in affected individuals [42].

**FIGURE 2:**
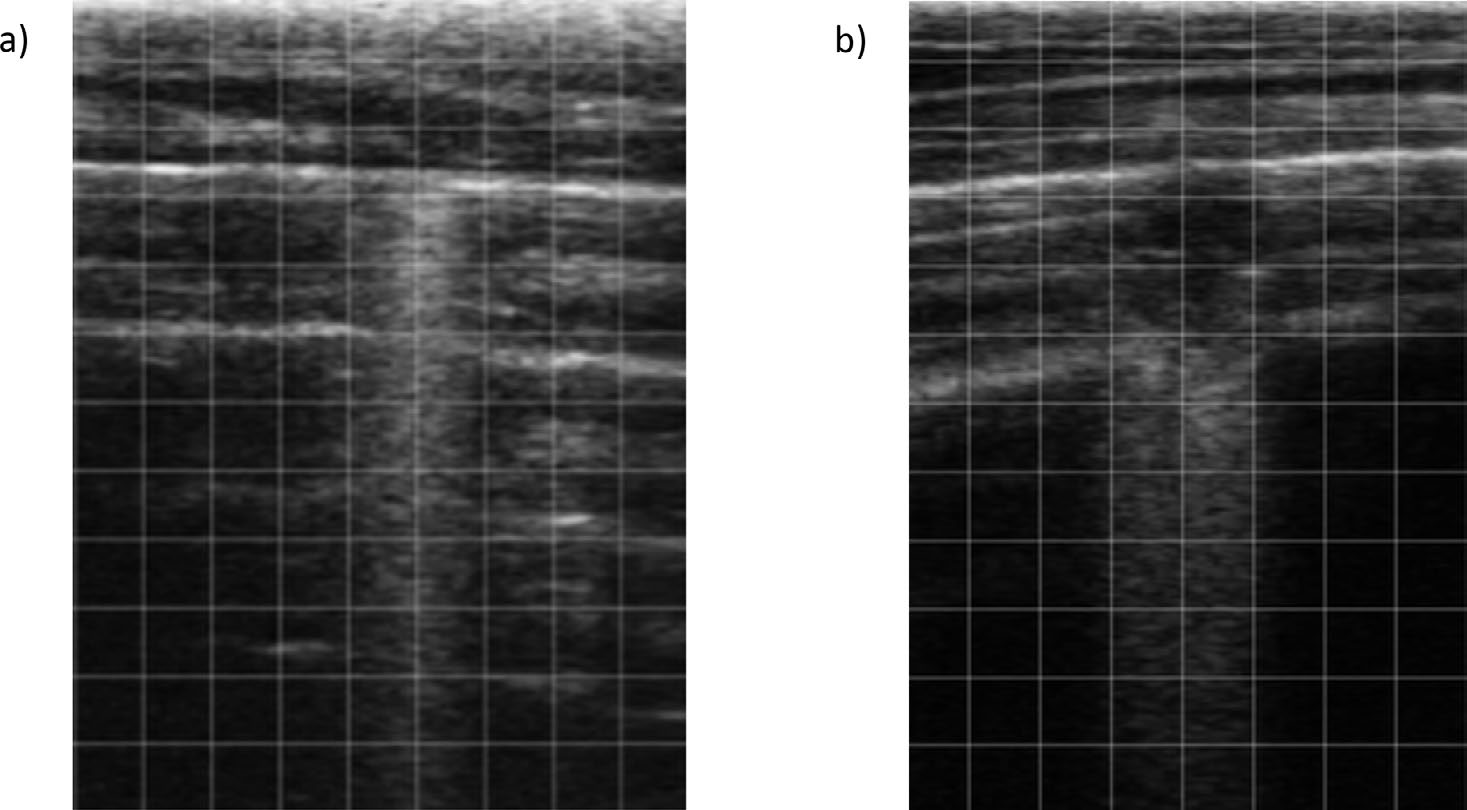
(a) Image of a comet tail artifact, with shadowing arising from the pleural surface; (b) image of a superficial alveologram. A focal hypoechoic region with acoustic shadowing radiating medially from the unaerated region is visible with disruption of the normal reverberation artifacts (A lines).

Discrete areas of consolidated lung are visible as (superficial) alveolograms, focal hypoechoic regions with acoustic shadowing radiating medially from the unaerated region [18]. Shadowing from these lesions should be distinguished from comet‐tails—where shadowing arises from the pleural surface, unlike the latter, where shadowing arises medial to the lesion (or “from the deep”) (Figure 2) [9, 43]. Progression of lung consolidation might be seen depending on the disease course [44]. The ultrasonographic definition of lung consolidation is associated with the replacement of air within alveolar space by liquid, inflammatory cells, and bacterial products. Various types of consolidation patterns can be seen depending on various factors, not limited to the interaction between calf immunity and the infectious pathogen, the presence of residual gas within the bronchi in the consolidated area, and the chronicity of existing lesions [18, 45]. However, this area deserves further research since almost all information on lung consolidation is based on quantitative assessment of lung consolidation extension.

Other types of lung and pleural anomalies are described, such as the presence of an abnormal amount of pleural fluid within the pleural space or cavitary lesions indicative of abscess formation in consolidated lung. Pneumothorax can also be diagnosed and monitored with thoracic ultrasonography, monitoring the lung point, which is the area where the visceral and parietal pleura are separated due to intrapleural air accumulation [21, 39].

#### 3.4.2 Scoring Methods

Several ways of assessing BRD associated lung lesions are reported. The main scoring methods focus on the extent of lung consolidation using either ordinal or dichotomous scales. By definition, if lung consolidation can be seen in a particular area, the specific region of lung parenchyma is not participating in gas exchange. A decrease in all metabolic pathways that are oxygen dependent (including those associated with growth) is likely. The extent is variable, and while alterations in respiratory gas exchange have been described in calves with severe lesions or chronic disease, under field conditions, minimal, clinically irrelevant changes in acid–base or oxygenation in calves with consolidation of ≥ 1 or ≥ 3 cm depth have been described [46, 47, 48, 49]. Nonetheless, lung lesions' extension is also associated with an increased risk of relapse or retreatment at first BRD pull in feedlot calves, with increased risk of death or early culling in dairy calves and with longer treatment durations and reduced likelihood of cure in veal calves [6, 32, 50, 51].

The main scoring methods are indicated in Table 1. The most commonly used ordinal scale is a 6‐level scale, which final scoring represents the higher level of anomalies found after bilateral scanning of the lung from the 10th to the 2nd intercostal space on the left side and from the 10th to the 1st intercostal space on the right side [20]. A score of 0 is attributed when no consolidation is found and when only a small number of comet‐tails are observed. A score of 1 is associated with examinations with diffuse B‐lines but no consolidation. A score of 2 is noted when lobular consolidation is observed. The definition of lobular consolidation can sometimes be an ambiguous term. It has been defined as small discreet areas of consolidation within an aerated lung lobe [52], which from a practical standpoint can be defined as a calf with an extent of consolidation between 1 and 3 cm of maximal depth [52]. A score of 3 is given to calves with 1 lung lobe with lobar consolidation (meaning that the full length of the lung is consolidated). This has also been associated with a consolidation depth of 3 or more centimeters [53]. Scores of 4 and 5 are given when lobar consolidation is found in 2 or 3 or more lung lobes, which requires that operators are able to distinguish specific lung lobes by the position of their ultrasound probe.

**TABLE 1:** A summary of published binary/ordinal scoring methods used during TUS of calves.

First author	Publication date	Scoring region(s)	Scoring criterion	Total score
Reinhold	2002	The lung is divided into craniodorsal, caudodorsal, cranioventral, caudoventral segments bilaterally	0 = Aerated lung 1 = Comet tails 2 = Echogenic pattern with consolidation < 1 cm depth 3 = Echogenic pattern + pleural reflective band or reverberation artifacts 5 = Echogenic pattern only	0–40
Ollivett	2011	Right: 10th—1st ICS Left: 10th—1st ICS	Abnormal lung was defined as ≥ 1 cm^2^ of non‐aerated lung	—
Ollivett	2016	Right: 10th—1st ICS Left: 10th—2nd ICS	0 = Normal aerated lung, no consolidation and none to few comet‐tail artifacts 1 = Diffuse comet tails without consolidation 2 = Lobular pneumonia, discreet consolidation ≥ 1 cm^2^ within an otherwise aerated lung 3 = Consolidation of an entire lung lobe 4 = Consolidation of two entire lung lobes 5 = Consolidation of three or more entire lung lobes	—
Adams	2016	3rd to 10th ICS bilateral	1 = Calves with no abnormalities, only a healthy pleural surface or an isolated comet tail within an image field 2 = Multiple comet tails without significant lung consolidation 3 = 1 or more consolidations > = 1 cm (depth?) 4 = ≥ 6 cm (depth?) consolidation, abscessation or pleural effusion > 1 cm	1–4
Tejero	2016	Right: 10th–1st ICS Left: 10th–2nd ICS	0 = < 1 cm consolidation 1 = 1 cm consolidation 2 = 2 cm consolidation 3 = 3 cm consolidation 4 = Lobar consolidation	0–4
Teixeira	2017	Right: 2nd–10th ICS Left: 3rd–9th ICS	NC = No consolidation LC = Any size lung consolidation	—
Abdallah	2019	Right: 10th–1st ICS Left: 10th–2nd ICS	Binary cut‐offs using 1 and 3 cm depth criterion	—
Cuevas‐Gomez	2020	Left: 4th–6th ICS Right: 4th–6th ICS	0 = Aerated lung 1 = ≥ 1 comet tail 2 = Consolidation ≥ 1 cm^2^	
Porter	2021	9 specific sites, 5 on the left and 4 on the right	Scores assigned individually to 9 sites: Pleural defects graded: 0 = No comet tails 1 = 1–2 comet tails 2 = 3–5 comet tails 3 = ≥ 5 comet tails Consolidation graded: 0 = No consolidation 1 = < 2 cm consolidation 2 = > 2 cm but < 4 cm consolidation 3 = > 4 cm consolidation Scores for each site added together to produce a final score based on total value: 0 = Consolidation grade 0 and pleural defects score < 5 1 = Consolidation grade 0 and pleural defects score > 5 2 = Consolidation grade 1 3 = Consolidation grade 2 4 = Consolidation grade 3	0–4
Akter	2021	Left: 3rd–12th ICS Right: 3rd–12th ICS	1 = Aerated lung, no comet tail artifacts 2 = Moderate consolidation: no more than 2 discrete areas of consolidation < 3 cm^2^, diffuse comet tails 3 = A single area of consolidation ≥ 3 cm^2^ or > 2 areas of consolidation < 3 cm^2^	
Fiore	2022	Left: 10th–7th ICS, 6th–5th ICS, 4th–3rd ICS Right: 10th–7th ICS, 6th–5th ICS, 4th–3rd ICS	In addition to recording of the total area of consolidation and 6‐point score [20], a lesion score was calculated for each of 6 lung areas: 0 = Absence of lesions 1 = Comet tails 2 = Hepatization 3 = Fluid alveolograms 4 = Comet tails and hepatization 5 = Comet tails and fluid alveolograms 6 = Hepatization and fluid alveolograms 7 = Comet tails, hepatization and fluid alveolograms Lesion scores were added together to produce a “global lesion score”	0–42
Massett	2022	Entire: Left: 10th–2nd ICS Right: 10th–1st ICS Cranial and middle: Left: 2nd–5th, 4th–5th ICS Right: 1st–5th, 3rd–5th ICS Cranial: Left: 2nd and 3rd ICS Right: 1st and 2nd ICS	Four‐point scale based upon lesion depth: 0 = None or consolidation < 1 cm 1 = consolidation of 1–3 cm 2 = consolidation of 3–5 cm 3 = consolidation > 5 cm Lesion width > 1 cm but < 1 cm depth scored as 0 to avoid misclassification with pleural abnormalities	0–3
Hoffelner	2023	Left = 10th–6th, 4th–2nd ICS Right: Diaphragm—1st ICS	Specific lesions were recorded and scores assigned: Comet‐tails and consolidation: 0 = no comet tails or consolidation 1 = > 5 comet tails 2 = > 10 comet tails or lobular consolidation 3 = lobar lesion of the entire lobe Alveolograms, Atelectasis and/or Pleural effusions were counted: 0 = Not present 1 = Present (assigned additively)	0–Inf
Elder	2023	Left: 10th–1st ICS Right: 10th–1st ICS	A 6‐point scoring method was performed [20]. Due to variation of lesion severity within the lobar score of 2, this score was not considered within the analysis. Healthy TUS scores were considered as those ≤ 1 whereas ≥ 3 was the threshold for positivity	
Boccardo	2024	Left: 10th–2nd ICS Right: 10th–1st ICS	0 = No consolidation or < 1 cm depth consolidation 1 = Diffuse comet tails 2 = Consolidation between 1 and < 3 cm 3 = Consolidation ≥ 3 cm depth	0–3
Hinnant	2024	Left: 7th–2nd ICS Right: 7th–1st ICS	Modified version of the scoring system described by Ollivett and Buczinski (2016): 0 = No to few comet tails, aerated lung 1 = Diffuse comet tail artifacts 2 = Consolidation ≥ 1 cm^2^ and up to 3 cm^2^ 3 = Consolidation > 1 cm in width and full thickness of 1 lobe 4 = Consolidation > 1 cm in width and full thickness of 2 lobes 5 = Consolidation > 1 cm in width and full thickness of ≥ 3 lobes	0–5
Jourquin	2024	Right: 8th/9th–1st ICS Left: 8th/9th–2nd ICS	Consolidation depth was measured and calves categorized as: Healthy = No consolidation Mild = Consolidation < 1 cm Moderate = Consolidation 1–3 cm Severe = Consolidation ≥ 3 cm	
Lisuzzo	2024	Left: 10th–3rd ICS Right: 10th–3rd ICS	Modified version of the scoring system described by Fiore et al. (2022). The lung fields are divided into six regions with a score assigned to each region: 0 = Healthy lung 1 = Comet tails 2 = “Spot” of lobular consolidation 3 = Lobar consolidation 4 = Lobar consolidation and comet tails 5 = Fluid alveologram/bronchogram 6 = Fluid alveologram/bronchogram and comet tails 8 = Lobar consolidation and fluid alveologram/bronchogram 9 = Lobar consolidation and fluid alveologram/bronchogram and comet tails 11 = Pleuritis The sum of the scores for all six regions is then calculated and disease positivity defined as a score > 10.5. The authors also used the 6‐point method described by Ollivett and Buczinski (2016)	
Rouault	2025	Left: 4th–5th ICS Right: 4th–5th ICS	Each ICS divided into ventral and caudal portions. The total number of portions with a consolidation was then calculated to produce a score (0–8)	
Feitoza	2024 and 2025	Right: 8th–11th ICS	Produced an ultrasound lung score: 1 = < 3 B lines (< 7 mm thick) 2 = ≥ 3 B lines (< 7 mm thick) 3 = B lines (> 7 mm thick) 4 = Multiple B lines (> 7 mm thick) with abnormal pleural findings 5 = Consolidated lung with pleural thickening/irregularities and effusion Also recorded A‐line and B‐line “count category” as a dichotomous variable based upon their frequency (0–2 and ≥ 3)	
Boccardo	2025	Right: 10th–1st ICS Left: 10th–2nd ICS	0 = No lesions or < 1 cm depth consolidation with comet tails 1 = Patchy lesions or consolidation between ≥ 1 cm and < 3 cm depth 2 = Consolidation ≥ 3 cm depth	
Jourquin	2024	Right: 8th/9th–1st ICS Left: 8th/9th–2nd ICS	Modified version of scoring system described by Jourquin et al. (2024): Mild = < 1 cm depth Moderate = 1–2.5 cm Severe = ≥ 3 cm	

*Note:* To avoid duplication, this list is not exhaustive. Instead, each scoring method is described once, and publications using previously published scoring methods are not described.

Abbreviations: ICS, intercostal space; TUS, thoracic ultrasound score.

Another common way to assess lung lesions is by measurement of consolidation depth or area. Maximal lesion depth is measured as a straight line originating at the pleural interface and running perpendicularly to it. To be measured accurately, operators must be confident in distinguishing consolidated lung from acoustic shadowing (Figure 2). Consolidation area is typically determined using a scanner with a grid function. This overlays a grid of a fixed size on top of the image. If the grid size is known, the number of grids with consolidated lung can be counted and the and area calculated (e.g., using a 1 cm width × 1 cm height grid function, each grid is equivalent to 1 cm^2^. Consolidation occupying two complete grids would be equivalent to 2 × 1 cm^2^ = 2 cm^2^). Imaging using TUS usually is usually performed via a 2‐dimensional image, and the reporting of area refers to a cross‐sectional measurement (i.e., cm^2^) rather than three‐dimensional volume (cm^3^). When large areas of lung are consolidated, measurement of area can be challenging, especially when the affected area extends beyond a single image frame. A more simplified approach measures if consolidation is higher or lower than a particular threshold that can either be associated with the maximal depth (e.g., > 0 cm, ≥ 0.5 cm, ≥ 1 cm, or ≥ 3 cm thresholds) or maximal area of consolidation (e.g., ≥ 1 cm^2^, ≥ 2cm^2^) [54, 55]. An intermediate suggestion is the use of a trichotomous scale to classify mild (< 1 cm), moderate (1–3 cm or 1–2.5 cm), and severe (≥ 3 cm) consolidation depths [6, 51, 56]. Other ultrasonographic scoring systems are reported less frequently and some account for other ultrasonographic anomalies such as pleural effusion or signs of pneumothorax (Table 1). Notably, while multiple studies have compared the accuracy of depth measures to identify BRD, the authors are aware of only a single manuscript comparing depth and area and none evaluating scoring methods. Therefore, at present, it is difficult to know which of these methods are more accurate to detect BRD [5, 57].

### 3.5 Intra‐ and Inter‐Operator Agreement

Operator factors are a key determinant of the accuracy of TUS, which is especially crucial to prevent unintended misuse of antimicrobials [58]. Both the performed scanning method and method of image interpretation will be affected by operator factors, and inter‐operator agreement is a crucial metric to understand and report. Eight publications reporting measures for agreement for TUS were identified. Agreement or reliability indicators reported in articles included in this review ranged between fair and moderate according to the authors' own benchmarks (Table 2), however, direct comparison remains challenging due to differences in the indicators used, the ultrasonographic scales applied, the skill levels of the participant, and variable disease prevalence within the study populations. Therefore, prospective studies specifically addressing this aspect are warranted. Overall, cross‐sectional measurements in both research and clinical settings will suffer from a relative under‐ or over‐reporting of disease if agreement is poor (and variability is high between or within operators), whereas longitudinal measurements are at risk of suggesting misleading outcomes. Consider, for example, if the effects of treatment responses or disease progression are falsely impacted by variability between operators rather than changes in the examined animals. In the context of TUS, intra‐operator agreement represents the variability of the same individual performing a consistent scanning technique and image interpretation. Practically, agreement will be affected by the complexity of the technique or outcome measure. Binary outcomes are inherently more likely to be associated with a greater amount of agreement, although a proportion of that will occur by chance. Measured in feedlot calves, operators of variable experience were reported to have good agreement and reliability for the identification of consolidation from TUS recordings, whereas a 6‐point scoring method demonstrated poorer agreement when compared to a binary one in TUS novices [11, 59]. Operator training is crucial, as agreement (and diagnostic sensitivity) improves with increasing expertise [58, 60, 61]. While the quality of TUS research will be affected by intra‐, inter‐, and rater reliability factors, of the 40 manuscripts considered in this article, of those papers not specifically investigating operator performance, only a single article described the aforementioned metrics [62]. It is also important to mention that there is not a gold standard way to report and measure intra‐and inter‐operator chance‐adjusted agreement, especially in contexts where the prevalence of anomalies is low [63]. The Cohen Kappa or other kappa‐like statistics can be severely compromised by the overestimation of agreement just by chance in these contexts, lowering these indicators despite high raw agreement [64].

**TABLE 2:** A summary of operator agreement metrics measured in the studies that were included as part of this review.

First author	Publication date	Assessment^a^		Criteria^b^	Inter‐operator agreement^c^	Intra‐operator agreement^d^
		Technique	Method			
Buczinski	2013	Y	Y	CD ≥ 1 cm	*κ* = 0.6–1.0	ICC *C* = 0.77–0.99 ICC *A* = 0.73–0.89
De Cremer^e^	2018	Y	Y	CD	PA = 78%–96% *κ* = 0.38–0.78	—
Buczinski	2018	N	Y	CD ≥ 1 cm CT	PA = 0.84 (0.80–0.89) F*κ* = 0.67 (0.49–0.86) AC1 = 0.68 (0.51–0.86)	—
				PI	PA = 0.82 (0.80–0.87) F*κ* = 0.56 (0.33–0.80) AC1 = 0.73 (0.58–0.89)	
				PE	PA = 0.62 (0.53–0.67) F*κ* = 0.20 (−0.05–0.44) AC1 = 0.21 (−0.01–0.44)	
				Overall	PA = 0.82 (0.75–0.86) F*κ* = 0.71 (0.51–0.92) AC1 = 0.71 (0.51–0.92) ICC = 0.52–0.70S	
Pardon^e^	2019	Y	Y	CD ≥ 1 cm	*κ* = 0.38–0.49	—
Cantor	2022	Y	Y	CA ≥ 3cm^b^	*Cκ* = > 0.90	—
Hoffelner	2023	Y	Y	CD, CT, AT, AL, PE	*κ* = 0.83–1.00	*κ* = 0.90–0.95
Jourquin	2024	Y	Y	CD ≥ 1 cm	*Cκ* = 0.40 (SD = 0.24) KA = 0.24 (0.20–0.27)	—
Lindley	2024	Y	Y	TUS6	PA = 0.31 (0.21–0.42) KA = 0.17 (0.05–0.21)	—
Lindley	2024	Y	Y	CA ≥ 3cm^b^	PA = 0.72 (0.59–0.86) KA = 0.36 (0.21–0.52)	—

^a^ Component(s) of TUS assessed as part of each study. Technique includes articles requiring operators to perform TUS on individual calves as part of the agreement assessment. Method includes articles requiring operators to interpret images as part of the agreement assessment. Y = yes, N = no.

^b^ Criteria for which agreement was calculated against. CD = consolidation depth, CA = consolidation area, CT = comet tail artifacts, PI = pleural irregularity, PE = pleural effusion, AT = atelectasis, AL = alveologram, TUS6 = a 6‐point scoring technique as described by Ollivett and Buczinski [20], and Overall = all quantitatively measured variable indicators.

^c^ *κ* = Kappa, F*κ* = Fleiss' Kappa, C*κ* = Cohen's Kappa, PA = percentage agreement, AC1 = Gwet agreement coefficient, KA = Krippendorf's alpha. Unless stated otherwise, where available, interquartile ranges for PA values and 95% CI for all other measures are provided in brackets.

^d^ ICC C = intraclass correlation coefficient for consistency, ICC A = intraclass correlation coefficient for agreement.

^e^ These publications appear to describe the same dataset. Since different inter‐operator values are reported, both are included for completeness.

### 3.6 Outcomes

Ultrasonographic findings have been associated with various health and production outcomes (Table 3). Among those reported, it is important to note that almost all studies focusing on these issues do not account for the pathogens causing the disease. The occurrence and development of consolidation will be partially determined by the infectious agent(s), as will prognostic factors and herd‐level implications [3, 65]. Complicated by the multifactorial, polymicrobial nature of BRD, the varied local and global diversity of pathogen virulence and serotype/genotype, a lack of rapid diagnostics, and the common use of empirical antimicrobial therapies, long‐term consequences will be affected by the etiology and epidemiology of the disease [66, 67, 68, 69]. For example, equivalent consolidation caused by monomicrobial infection with M*ycoplasmopsis bovis* will is likely to have more severe and longer‐lasting consequences than disease caused by *Pasteurella multocida* , whereas synergistic polymicrobial infection might be the most severe [70, 71, 72, 73]. With improvements in rapid diagnostic techniques for BRD, opportunities exist for researchers to investigate these associations more comprehensively, and where possible, the reporting of pathogen identification measures alongside TUS outcomes could improve the context in which findings are interpreted [6, 74, 75, 76].

**TABLE 3:** A summary of production outcomes measured in the studies, which were identified as part of this review.

Production outcome	No. of studies	First author	Publication date
Average daily gain	9^+^	Tejero	2016
		Abdallah	2019
		Timsit	2019
		Cramer	2019
		Binversie	2020
		Cuevas‐Gómez	2020
		Rhodes	2021
		Cuevas‐Gómez	2021
		Saadatnia	2023
		Jourquin^d^	2023
		Lisuzzo	2024
		Hoffelner	2024
Reproductive indices^b^	2	Teixeira	2017
		Baxter‐Smith	2022
Milk production^c^	1	Dunn	2018
Survival	2	Adams	2016
		Baxter‐Smith	2022

*Note:* In each category, studies that identified a statistically significant negative production outcome associated with lung lesions identified by thoracic ultrasound.

^a^ Average daily gain: The period over which ADG was measured was not consistent across the studies.

^b^ Age at first calving, pregnancy hazard postpartum.

^c^ First lactation 305 day milk yield.

^d^ Authors also reported growth in terms of cold carcass weights.

After inoculation of the lower respiratory tract with pathogenic bacteria, the onset of lung consolidation can be rapid. After experimental infection with *Mannheimia haemolytica* , exudate‐filled infiltrates were identified as soon as 2–3 h later [44]. Changes in feeding and attitude as well as hematological and biochemical factors occur, although their effectiveness for identifying consolidation is variable but generally low unless concomitantly associated with clinically identifiable outward signs of disease [49, 77, 78, 79, 80, 81]. In the long term, increased risk of early culling, decreased reproductive performance, decreased milk production, reduced daily liveweight gain, and carcass quality have been reported in calves with ultrasonographic lung consolidation [10, 11, 25, 35, 82, 83, 84, 85, 86, 87]. Risk of clinical relapse or failure of cure might also be attributed to consolidation severity or chronicity [6, 88, 89]. Limitations to these comparisons exist. Comparisons between studies are challenging due to population and etiology differences, various protocols used for scanning the calves, different times of examination and treatment history as well as the different criteria used to define abnormal thoracic ultrasound findings. For example, reaeration of a consolidated lesion might be a useful indicator of cure. However, the extent of reaeration after treatment and its association with risk of relapse is variable. In beef cattle, reaeration of consolidated lesions to < 0.5 cm depth was a useful cure criterion, whereas in veal calves the same authors found regression < 1 cm to be ineffective [6, 26]. Low cure rates in the latter study contrast with extremely high rates of aeration in another veal‐calf setting [33]. The continued integrated use of TUS alongside diagnostics within various settings offers the opportunity to provide specific recommendations to populations accounting for pathogen and environmental factors.

The implications of lung consolidation severity will also be affected by animal factors. Measures of consolidation depth or area, particularly when based on binary cut‐offs, fail to account for differences in the proportion of total lung volume affected. For instance, the consequences of a 3 cm depth consolidation in a neonatal calf with smaller lungs could be greater than compared to a larger, older animal with greater lung volume and a smaller relative proportion of consolidation. At present, this effect is not accounted for. To improve comparability across animals of different sizes, in the future a ratio‐based measure incorporating body or lung size could be used. In human pediatrics, standing height strongly correlates with lung growth and function, as measured by spirometry, suggesting that withers height could serve as a similar metric in animals if validated [90]. Alternatively, heart size metrics, such as diastolic left ventricular internal diameter or intraventricular septal width, which are readily obtainable during TUS, could provide a practical measure allowing improved comparability between varying animal and consolidation sizes. Currently, no studies have integrated these measures into TUS assessments of lung consolidation severity, and their incorporation will increase examination time and complexity, potentially reducing its feasibility. Furthermore, growth of lung and heart tissue as well as skeletal development associated with height gain is not linear but allometric, underscoring the need for further research to evaluate their practical utility. Ultimately, approaches which seek to standardize such animal factors and account for production system and pathogen involvement are necessary.

## 4 Other Considerations

### 4.1 Cohort Differences

The production context from which TUS is performed will have a crucial influence on the interpretation of any generated outcome measures. From the relevant publications considered in this manuscript, TUS has been applied across a variety of cattle types including dairy (57%, *n* = 27), beef (26%, *n* = 12), and veal (17%, *n* = 8) calves. Age differences will also contribute to parities, and of the research considered in this manuscript, ages ranged from newborn calves up to 10 months of age, although a number of studies did not disclose age information, which was likely higher. In well‐conditioned calves, less experienced operators might struggle to locate the intercostal spaces and maintain alignment. The muscle cover in highly conditioned beef calves might also limit the extent to which an operator can scan the cranial portions of the lung. In extreme cases, the thoracic wall musculature can be deep enough that a reverberation artifact cannot be generated, and it might not be possible to determine if the lung parenchyma below is aerated. This is an unlikely scenario and can vary depending on the specification of the ultrasonography machine being used. Although the above factors might pose a challenge, TUS has been performed successfully in weaned continental breeds of beef cattle [54]. Nevertheless, it is likely that the sensitivity of TUS at diagnosing consolidation in well‐conditioned beef cattle is reduced in comparison to dairy or veal calves, especially if undertaken by an inexperienced operator. Readers and operators should interpret the results of TUS and study outcomes within the context of the population they were conducted in. Additionally, the diagnostic performance of TUS is likely to vary across these populations, not only due to phenotypic variation but also due to a pathogen and environmental differences as well as due to differences in disease prevalence, which will affect its negative and positive predictive value. These might be epidemiological, geographic, or temporal, associated with variation in pathogen presence, load, and infectivity, or with factors influencing transmissibility (not limited to housing environment and density, concomitant stressors, and comingling) [29, 69].

### 4.2 Limitations of TUS

Limitations do exist to the use of TUS as a diagnostic tool. Although observable changes to lung parenchyma can be visualized rapidly after the onset of lower respiratory tract disease, determination of lesion chronicity is more challenging [88]. Fibrosis, atelectasis, or lung infarction can have an ultrasonographic appearance similar to that of consolidation, and although disease indicators such as fibrin could be suggestive of a more chronic disease process, these are observed inconsistently [20]. As a consequence, TUS has imperfect sensitivity and specificity to detect active bronchopneumonia, which can complicate decisions regarding the treatment‐worthiness of individuals. Notably, the presence of consolidation is an imperfect proxy for the presence of an infectious bacterial bronchopneumonia requiring antibiotic therapy. Notably, whilst a relationship between consolidation depths of ≥ 0.5 and ≥ 1 cm and pneumonia of a bacterial etiology has recently been demonstrated, another study associated consolidation with bacterial or viral presence [23, 30, 91, 92].

Due to air impedance, thoracic ultrasonography is only able to detect chest‐wall lesions. Subpleural consolidation or abscessation surrounded by unaffected tissue is not visible and will be missed, as will pulmonary bullae, present as gas‐containing cystic‐like structures produced by confluent destruction of adjacent alveolar walls [4, 39, 93, 94].

The etiology of respiratory disease cannot be inferred using TUS alone, although it is possible to recognize and refine inflammatory lesion distribution phenotypes. Interstitial pneumonia associated with lung edema can be recognized by the presence of diffuse comet tails, whereas localized increased parenchyma hyperechogenicity can be suggestive of granulomatous or fibrinous lesions. Regional distribution can also be suggestive of the disease process. Verminous (parasitic) pneumonia is typically associated with a caudodorsal distribution, whereas infectious bronchopneumonia most commonly locates within the cranioventral lobes. Although it has been described pathologically, there is no evidence to suggest that an interstitial pattern recognized on TUS can be used to infer a viral etiology [95]. Differences in lesion presentation and localization might reduce the sensitivity of TUS, dependent upon diagnostic methods and operator vigilance.

### 4.3 Caveats of Scoring Methods

**FIGURE 3:**
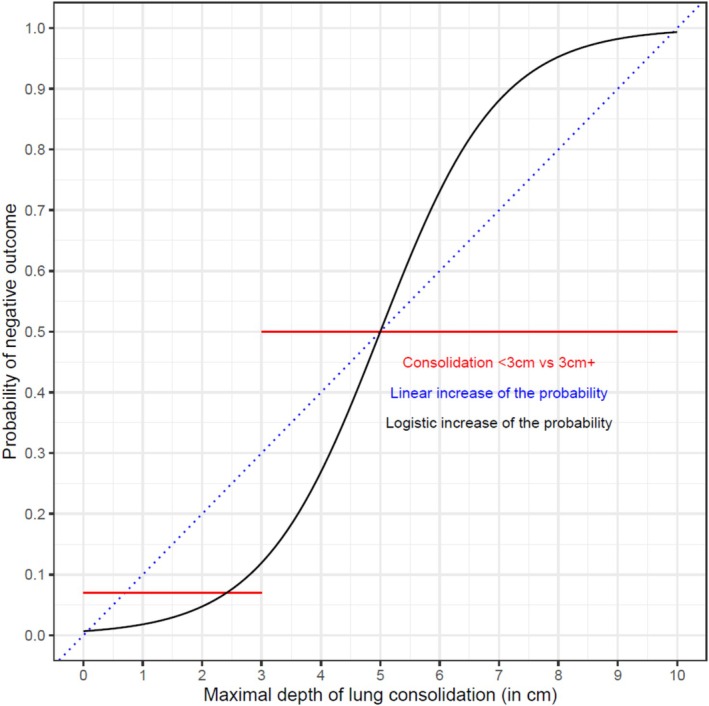
Illustration of the different assumptions of scoring systems using a threshold for determining negative outcomes. A linear relationship between maximal depth of consolidation (probability of negative outcome increase linearly for each additional centimeter of maximal depth) is presented (blue dotted line). For most dichotomous outcomes (survival, relapse (Y/N)), the increased risk is generally represented as a logistic curve (black line) since assuming a linear relation of the predictor of interest (here consolidation depth) and the log‐odds of the probability of the negative outcome. Therefore, the increased probability is not linearly associated with the depth of consolidation. When using a “threshold approach” (here deciding that 3 cm or more of consolidation are associated with negative outcomes), we assume that the probability outcome of is the “mean” risk of the strata with the consolidation range. In this particular case, we can obviously see, for example, that a calf with 8 cm depth of consolidation would have a predicted probability of negative event at 0.95, whereas it would be estimated at only 0.5 when using the dichotomous approach. On the other hand, a calf with 3 cm consolidation depth will still be estimated at a 0.5 probability of negative outcome with the dichotomous approach versus 0.12 using the continuous logistic curve approach.

A key advantage of scoring methods or dichotomous thresholds is that they might easily be understood by producers and key stakeholders. However, there is a risk of oversimplification, especially when attempting to understand the effect of consolidation on studied outcomes [96]. Categorization assumes that all animals in the same categories have the same outcome probability. As illustrated in Figure 3 underestimation of the true risk of a negative outcome in a calf just below the threshold (let's say a calf #1 with a maximal depth of 2.9 cm if using a 3 cm depth threshold) and, by contrast, overestimation of negative outcome probability in a calf #2 with consolidation just above the decision threshold since a calf with a maximal consolidation depth of 3.2 cm would be quoted as having the same probability of negative outcome as a calf #3 with 5.5 cm depth. However, the difference between calf #1 and #2 should be considered smaller than that between calf #2 and #3. This problem is inherent to continuous data categorization, and choosing the “best” cut‐off is a challenging task, especially since this might be affected by other animal factors such as size, age, and total lung volume [97]. A compromise might involve the use of trichotomous or other ordinal scales based on disease severity, which might offer a balance of simplicity and data granularity. On the other hand, even using a continuous approach would not be without limitations. Consolidation per se only means that a part of the lung is filled by other things than air. Depending on the pathophysiology generating consolidation and the etiology of bronchopneumonia, there could be a different meaning of a particular consolidation area or depth. However, this type of information is currently lacking.

Many scoring techniques do not specify the location of above‐threshold consolidation, nor do they account for the amount of scanned lung with above‐threshold consolidation present. For example, a calf with a localized lobular consolidation of 3.1 cm would be classified similarly to a calf with lobar consolidation ≥ 3 cm of multiple lobes or a calf with ≥ 3 cm depth in different intercostal spaces for the same lung lobe. To account for this, some authors have used ordinal scoring systems incorporating for consolidation depth and site [98]. Alternatively, the method proposed by Ollivett and Buczinski does partially account for severity based upon the extent of lobar or lobular consolidation occurring [20]. Nonetheless, this can also be practically challenging. Identification of lung lobes during TUS is reliant on the operator having a precise knowledge of the anatomical location of their probe, anatomical landmarks visible via TUS, and their relationship with each lung lobe. This might be challenging for practitioners performing TUS infrequently, although it allows the operator to accurately recommence scanning if interrupted by animal movement during the exam. Alternatively, if knowledge of the exact lobes affected is not crucial, location might be described more simply; for example, the caudal aspect of the heart base is an easy anatomical landmark, and authors might describe lesions by their relative location, either cranial or caudal to it [23, 51].

Ordinal and dichotomous scoring systems also present a similar challenge when determining if treatment has been successful, with variable criteria used to define treatment success or failure. Reaeration, the reappearance of reverberation artifacts in previously consolidated regions of lung, has been suggested as an indicator of cure [26]. However, different published thresholds have been used to define “reaeration”. For example, consolidation ≥ 0.5 cm has been used as a criterion for pneumonia, and complete aeration of previously consolidated lung as cure [26]. More recently, regression of consolidated tissue ≥ 1 cm depth to < 1 cm has been used to define cure, albeit with a high frequency of consolidation reoccurrence in cases without complete aeration [86]. Factors associated with the risk of relapse and likelihood of self‐cure remain to be elucidated, and it is not clear if using a scale that has been developed for diagnosis is appropriate when determining treatment success.

Ultimately, no scoring or measurement‐based method is able to perfectly characterize every lesion encountered. Nonetheless, recognition of their caveats is essential. Future studies specifically designed to look for the diagnostic and prognostic accuracy of different ways to score and assess lung lesions using ultrasonography are therefore necessary.

### 4.4 How Do We Achieve Consensus?

Achieving a consensus could be obtained by parallel actions. First, comparing the diagnostic and prognostic accuracy of the different ways to assess lung lesions and finding a trade‐off between accuracy (proportion of animals accurately classified by the test) and reliability (ability of the test conducted by different raters in different settings to detect variation between animals beyond sources of measurement errors). An almost perfectly accurate way to assess lung lesions would be useless if not practically feasible in the field by a veterinary practitioner with a minimal specific lung ultrasound background. Secondly, experts could also a priori select a set of possible scoring methods that could be chosen based on their applicability and potential to be applied routinely in the field. Using this approach might be a good compromise between evidence‐based and practice‐based approaches. Ultimately, the objective is to broaden the applicability of lung ultrasound to tailor respiratory disease management and to give another objective tool that can be used to monitor respiratory health in cattle. For uptake to be maximized, the relative ease to learn, perform, interpret, and report data is crucial, especially since a lack of confidence has been identified as a major barrier to the implementation of point‐of‐care ultrasound within small animal practice [99]. Within human medicine, practitioner consensus has led to the development of standardized models for the reporting of TUS, whilst regulated and accredited training courses ensure high‐quality examinations are performed by all practitioners [100, 101, 102]. Similar initiatives, such as the Register of Mobility Scorers (ROMS) for bovine locomotion scoring, have improved the national reporting of lameness in UK dairy herds [103]. Within the field of bovine TUS, the authors are aware of only a single training and certification course in thoracic ultrasound [58]. Effective training and certification, as well as consistent reporting of TUS findings, will improve the accessibility of TUS and its research outcomes, allow easy data compilation, direct clinical research, and maximize benefit to all stakeholders.

## 5 Conclusion

Thoracic ultrasonography is a fast, relatively simple calf‐side diagnostic technique. Its use, principally described for the detection of lung consolidation associated with bronchopneumonia, provides an accessible, low‐cost solution that can improve the precision of the diagnosis of respiratory disease. The implementation of standardized measurement and reporting of TUS offers an opportunity to improve research transparency, refine our understanding of the long‐term implications of lung consolidation, and recognize research gaps. An appreciation of the current techniques for performing TUS and grading lesion severity, their caveats, and challenges with their implementation is essential for stakeholders in a field that is rapidly increasing in popularity.

## Disclosure

Authors declare no off‐label use of antimicrobials.

## Ethics Statement

Authors declare no institutional animal care and use committee or other approval was needed. Authors declare human ethics approval was not needed.

## Conflicts of Interest

George Lindley has previously been a paid speaker by Krka. Sébastien Buczinski has previously been a paid speaker by Zoetis, MSD, Hipra, EI Medical, Ceva and Vetoquinol. John Donlon has previously been paid speaker honoraria by Boehringer, Bimedia, MSD and Zoetis. These parties had no role in the study design, interpretation or decision to submit this manuscript for publication.

## Supporting information


**Data S1:** Supporting Information.
