# Supplementary material for: Thoracic Ultrasonography in Calves: A Narrative Review of Techniques and Reporting Practices
Source: J Vet Intern Med. 2025 Sep 30;39(6):e70251. doi: 10.1111/jvim.70251 (PMC12483840; doi:10.1111/jvim.70251)
Supplement: Supplementary file 1 — Data S1: Supporting Information. [file JVIM-39-e70251-s001.docx]

TABLE S1 List of eligible papers for consideration after screening and their criteria for retention

| No.^1^ | Type^2^ | No. of calves^3^ | No.  Exams^4^ | Age^5^ | Breed^6^ | Sex^7^ | Production system^8^ | Country^9^ | Interpretation method^10^ | Scoring scale^11^ | Production outcomes^12^ | Operator^13^ | Inter-operator agreement^14^ | Intra-operator agreement^15^ | Reason for inclusion^16^ |
| --- | --- | --- | --- | --- | --- | --- | --- | --- | --- | --- | --- | --- | --- | --- | --- |
| ^1^ | FP | 17 | 34 | 2-3m | XB | ND | NA | Germany | S | 0-20 | ND | ND | ND | ND | II |
| ^2^ | FP | 66 | 66 | 0-14d | O | ND | NA | Germany | M | ND | ND | ND | ND | ND | T |
| ^3^ | SC | 10 | 30 | >7d | DB | M | Veal | Canada | D, CT, PE | ND | ND | 2 x N,  1 x Exp | Y | ND | A |
| ^4^ | SC | 239 | 239 | 3m | J | F | Dairy | Canada | S | 1-4 | S | ND | ND | ND | O |
| ^5^ | FP | 25 | ND | 1-3m | H | M | NA | Canada | CT, D, S | 0-4 | ND | ND | ND | ND | T, II |
| ^6^ | FP | 174 | ND | ND | ND | M, F | Feedlot | Canada | D, PE, S | ND | ADG | ND | ND | ND | T, II |
| ^7^ | C | 1066 | ND | <2m | ND | F | ND | Spain | D, S | 1-4 | ADG | ND | ND | ND | O |
| ^8^ | FP | 613 | 613 | 60d | H | F | Dairy | USA | S | 0-1 | R, S | ND | ND | ND | O |
| ^9^ | FP | 210 | 50^*^ | ND | ND | M, F | Feedlot | Canada | M | ND | ND | 2 x N, 1 x Adv, 1 x Exp | Y | ND | A |
| ^10^ | C | 50 | 50 | 8-12wks | ND | ND | Veal | Belgium | D | ND | ND | 2 x N, 1 x Exp | Y | ND | A |
| ^11^ | FP | 144 | 144 |  | ND | M, F | Feedlot | Canada | D, A, CT, PE | ND | ADG | ND | ND | ND | O |
| ^12^ | FP | 57 | 57 | 1-91 | H | F | Dairy | Canada | D | ND | ND | ND | ND | ND | O |
| ^13^ | FP | 30 | 180 | 7m | H | M | Feedlot | USA | P | 0-100 | ND | ND | ND | ND | II |
| ^14^ | FP | 233 | ND | 2-4wks | H | F | Dairy | USA | S, A, CT | 0-5 & 0-1 | ADG | ND | ND | ND | O |
| ^15^ | CP | ND | ND | ND | ND | ND | ND | ND | DT | ND | ND | 2 x N, 1 x Exp | ND | ND | II, A |
| ^16^ | FP | 196 | ND | 3-52d | H | M, F | Dairy | USA | S, Sub | 0-6 | ADG, TR, | ND | ND | ND | O |
| ^17^ | FP | 153 | ND | 6-8m | AA, HE, CH, LIM | M, F | Beef | Ireland | S, A, CT, Sub | 0-2 | ADG | ND | ND | ND | O |
| ^18^ | SC | 135 | 270 | 1-6m | HF | M, F | Dairy | Italy | S | 0-5 | ND | ND | ND | ND | T |
| ^19^ | FP | 62 | ND | 4wks | H | M, F | NA | USA | S, CT, D | 0-4 | ND | 2 x N | ND | ND | II, T |
| ^20^ | FP | 317 | 966 | 1d – 4m | DB, XB | M, F | Dairy | Ireland | S, CT, D | 0-5 | ADG | ND | ND | ND | O |
| ^21^ | SC | 347 | 347 | 21-61d | ND | ND | Dairy | UK | S, CT, D | 0-5 | ND | ND | ND | ND | T |
| ^22^ | FP | 347 | 347 | 21-61d | ND | ND | Dairy | UK | S, CT, D | 0-5 | R, S | ND | ND | ND | O |
| ^23^ | FP | 120 | ND | 0-3m | ND | M, F | Dairy | USA | D, Sub^α^ | ND | ND | ND | κ > 0.9 | ND | A |
| ^24^ | FP | 130 | 780 | 1-9m | BB | ND | Beef | Belgium | D, Sub | ND | ND | ND | ND | ND | T |
| ^25^ | FP | 300 | 300 | 1-2m | H, XB | M, F^θ^ | Veal | France | S, D | 0-3 | ND | ND | ND | ND | T |
| ^26^ | FP | 622 | 1222 | 2-4m | ND | ND | Veal | Austria | CT, S, PE | 0-6 | ND | 2 | Y | Y | A, II |
| ^27^ | FP | 221 | 1768 | 0-56d | ND | F | Dairy | Iran | D | 0-3 | ADG | ND | ND | ND | O |
| ^28^ | FP | 60 | ND | 8-12m | LIM | M | Beef | Italy | S, A | 0-5,  0-42 | ND | ND | ND | ND | T |
| ^29^ | FP | 29 | 58 | 1-11wks | H | F | Dairy | USA | S, Sub | 0-5 | ND | ND | ND | ND | II |
| ^30^ | SC | 23 | ND | 2wks-4m | ND | ND | Dairy, veal | Belgium | D | ND | ND | 38 x N  2 x Exp | Y | ND | A |
| ^31^ | C | 40 | ND | 6-8wks | HF | ND | Dairy | UK | S, A | 0-5 | ND | 8 | Y | ND | A |
| ^32^ | FP | 215 | ND | 3d-12wks | H | F | Dairy | Canada | D | ND | MP | ND | ND | ND | O |
| ^33^ | FP | 55 | ND | 1m-11y | O | M,F | NA | Austria | M | ND | ND | ND | ND | ND | M |
| ^34^ | RP | NA | NA | NA | NA | NA | NA | NA | M, S | 0-5 | ND | ND | ND | ND | II, T |
| ^35^ | C | NA | NA | NA | NA | NA | NA | NA | S | 0-5 | ND | ND | ND | ND | II |
| ^36^ | FP | 53 | ND | 1-4m | XB | M | NA | Ireland | A, Sub, S | 1-4 | ADG | ND | ND | ND | II, O |
| ^37^ | C | 23 | ND | 2wks-4m | ND | ND | Dairy, veal | Belgium | D | ND | ND | ND | ND | ND | A |
| ^38^ | FP | 444 | ND | 2-10wks | HF | M | Veal | Belgium | D, CT, PE, Sub, S | ND | CCW | ND | ND | ND | O, II |
| ^39^ | RP | NA | NA | NA | NA | NA | NA | NA | M | ND | ND | ND | ND | ND | T |
| ^40^ | FP | 305 | 1265 | 1-6m | H, AA | ND | Beef & Dairy | Brazil | D | 0-1 | ND | 2 x Exp | ND | ND | T |
| ^41^ | FP | 96 | ND | 1-3m | HF, IS, R, XB | ND | Veal | Italy | S, A, D, CT, M | 0-66^•^ | ADG, TR | ND | ND | ND | II |
| ^42^ | FP | 600 | 1200 | ND | ND | M, F | Veal | Austria | CT, S, PE | 0-6 | ND | ND | ND | ND | O |
| ^43^ | FP | 221 | ND | 9m | CH, LIM, XB | M | Beef | France | A, S | 0-8 | ND | ND | ND | ND | T, O |
| ^44^ | FP | 62 | 62 | ND | ND | M, F | Beef | USA | S | 1-5 | ND | 1 x trained | ND | ND | T |
| ^45^ | FP | 819 | 819 | ND | ND | M, F | Beef | USA | S | 1-5 | TR, S | 1 x trained | ND | ND | II |
| ^46^ | FP | 86 | 86 | ND | ND | M, F | Dairy, beef, veal | Belgium | D | ND | ND | ND | ND | ND | II |
| ^47^ | FP | 1090 | 3256 | ND | ND | M | Veal | Belgium | D, S | 1-3 | TR, ADG | 4 x Exp | ND | ND | II, O |

^1^ Article number, as provided in the list of references.

^2^ Study type defined as either: FP = full paper, SC = short communication, C = conference proceedings, RP = review paper

^3^ Number of calves included in the study (after losses to follow-up).

^4^ Only included if provided or easily calculated. ND = no data, NA = not applicable.

^5^ If given in months it is marked with an “m” whereas if in days it is marked with a “d” and in weeks it is marked with “wks”. In some circumstances an approximate age range is described based upon the data presented within the manuscript.

^6^ H = Holstein, HF = Holstein-Friesian, J = Jersey, XB = Crossbreed, AA = Aberdeen Angus, HE = Hereford, CH = Charolais, LIM = Limousin, BB = Belgian blue, IS = Italian Simmental, R = Rendena, O = Other, DB = Unspecified dairy breeds, ND = no data.

^7^ F = female, M = male.

^8^ Production system where the study is performed. D = dairy, V = veal, B = beef, NA = not applicable (e.g. experimental facilities).

^9^ USA = United States of America, NA = not applicable

^10^ S = scoring, D = depth, A = area, CT = comet-tails, PE = pleural effusion, P = estimated percentage consolidation, Sub = bovine respiratory disease subtype, DT = Decision-tree, Articles recording additional information were labelled as, M = “medical” approach. See Table 1 for a summary of published binary/ordinal scoring methods used.

^11^ The scale of any described scoring method. NA = not applicable, ND = Not disclosed or available.

^12^ Reported outcomes relevant to production. Acute outcome measures were not recorded. ADG = Average daily liveweight gain, TR = treatment response, S = survival, R = reproduction, MP = milk production, CCW = cold carcass weight.

^13^ Reported level of expertise of the operators. N = Novice, Adv = experienced/advanced, Exp = expert

^14^ Y = Inter-operator agreement reported. ND = no data.

^15^ ND = no data.

^16^ A = operator agreement, II = method of image interpretation, O = outcome data, T = technique,

^*^ 50 videos were selected for appraisal out of 210 performed examinations.

^α^Disease positivity was defined as cases positive using parallel testing with ultrasound and clinical scoring.

^θ^Described by the authors as predominantly male.

^•^A 9-point scale is described with the scores 7 and 10 omitted from the scale to produce a value ranging 0-11 for each of 6 lung regions, with the total score then calculated as their sum.

List of References

1. Reinhold P, Rabeling B, Günther H, et al. Comparative evaluation of ultrasonography and lung function testing with the clinical signs and pathology of calves inoculated experimentally with Pasteurella muftocida. Vet Rec 2002;150:109-114.

2. Jung C, Bostedt H. Thoracic ultrasonography technique in newborn calves and description of normal and pathological findings. Vet Radiol Ultrasound 2004;45:331-335.

3. Buczinski S, Forté G, Bélanger AM. Short communication: Ultrasonographic assessment of the thorax as a fast technique to assess pulmonary lesions in dairy calves with bovine respiratory disease. J Dairy Sci 2013;96:4523-4528.

4. Adams EA, Buczinski S. Ultrasonographic assessment of lung consolidation postweaning and survival to the first lactation in dairy heifers. J Dairy Sci 2016;99:1465-1470.

5. Ollivett TL, Caswell JL, Nydam DV, et al. Thoracic Ultrasonography and Bronchoalveolar Lavage Fluid Analysis in Holstein Calves with Subclinical Lung Lesions. J Vet Intern Med 2015;29:1728-1734.

6. Abutarbush SM, Pollock CM, Wildman BK, et al. Evaluation of the diagnostic and prognostic utility of ultrasonography at first diagnosis of presumptive bovine respiratory disease. Can J Vet Res 2012;76:23-32.

7. Tejero C, Bach A. The hidden cost of a hidden disease: growth performance of calves as affected by bovine respiratory disease diagnosed using ultrasonography. J Anim Sci 2016;94:48-48.

8. Teixeira AGV, McArt JAA, Bicalho RC. Thoracic ultrasound assessment of lung consolidation at weaning in Holstein dairy heifers: Reproductive performance and survival. J Dairy Sci 2017;100:2985-2991.

9. Buczinski S, Buathier C, Bélanger AM, et al. Inter-rater agreement and reliability of thoracic ultrasonographic findings in feedlot calves, with or without naturally occurring bronchopneumonia. J Vet Intern Med 2018;32:1787-1792.

10. De Cremer L, Van Wissen M, Nelson L, et al. Interobserver agreement of a quick-scan lung ultrasound method in calves. In: 50th National congress of the Italian Association for Buiatrics; General meeting of the European College of Bovine Health Management 2018.

11. Timsit E, Tison N, Booker CW, et al. Association of lung lesions measured by thoracic ultrasonography at first diagnosis of bronchopneumonia with relapse rate and growth performance in feedlot cattle. J Vet Intern Med 2019;33:1540-1546.

12. Abdallah AA, Abdelaal AM, El-Sheikh AR, et al. Determination of the dynamics of respiratory diseases using thoracic ultrasonographic examination in preweaned dairy calves. Can Vet J 2019;60:859.

13. Baruch J, Cernicchiaro N, Cull CA, et al. Performance of multiple diagnostic methods in assessing the progression of bovine respiratory disease in calves challenged with infectious bovine rhinotracheitis virus and Mannheimia haemolytica. J Anim Sci 2019;97:2357-2367.

14. Cramer MC, Ollivett TL. Growth of preweaned, group-housed dairy calves diagnosed with respiratory disease using clinical respiratory scoring and thoracic ultrasound - A cohort study. J Dairy Sci 2019;102:4322-4331.

15. Pardon B. A quick scan lung ultrasound method and flow chart as a decision aid for bronchopneumonia diagnosis. In: XXIV congreso internacional ANEMBE de medicina bovina 2019;258-260.

16. Binversie E, Ruegg P, Combs D, et al. Randomized clinical trial to assess the effect of antibiotic therapy on health and growth of preweaned dairy calves diagnosed with respiratory disease using respiratory scoring and lung ultrasound. J Dairy Sci 2020;103:11723-11735.

17. Cuevas-Gómez I, McGee M, McCabe M, et al. Growth performance and hematological changes of weaned beef calves diagnosed with respiratory disease using respiratory scoring and thoracic ultrasonography. J Anim Sci 2020;98:skaa345.

18. Pravettoni D, Buczinski S, Sala G, et al. Short communication: Diagnostic accuracy of focused lung ultrasonography as a rapid method for the diagnosis of respiratory disease in dairy calves. J Dairy Sci 2021;104:4929-4935.

19. Porter MM, McDonald PO, Slate JR, et al. Use of thoracic ultrasonography to improve disease detection in experimental BRD infection. Frontiers in Veterinary Science 2021;8:763972.

20. Rhodes V, Ryan EG, Hayes CJ, et al. Diagnosis of respiratory disease in preweaned dairy calves using sequential thoracic ultrasonography and clinical respiratory scoring: Temporal transitions and association with growth rates. J Dairy Sci 2021;104:11165-11175.

21. Baxter-Smith K, More J, Hyde R. Use of thoracic ultrasound on Scottish dairy cattle farms to support the diagnosis and treatment of bovine respiratory disease in calves. Vet Rec 2022;190:e939.

22. Baxter-Smith K, More J, Hyde R. Associations between TUS scores and mortality and productivity outcomes on Scottish dairy farms. Cattle Pract 2022;30:34-35.

23. Cantor M, Renaud DL, Neave HW, et al. Feeding behavior and activity levels are associated with recovery status in dairy calves treated with antimicrobials for Bovine Respiratory Disease. Sci Rep 2022;12:4854.

24. Jourquin S, Bokma J, De Cremer L, et al. Randomized field trial comparing the efficacy of florfenicol and oxytetracycline in a natural outbreak of calf pneumonia using lung reaeration as a cure criterion. J Vet Intern Med 2022;36:820-828.

25. Masset N, Assié S, Herman N, et al. Ultrasonography of the cranial part of the thorax is a quick and sensitive technique to detect lung consolidation in veal calves. Vet Med Sci 2022;8:1229-1239.

26. Hoffelner J, Peinhopf-Petz W, Wittek T. Diagnostic and Prognostic Value of Clinical Scoring and Lung Ultrasonography to Assess Pulmonary Lesions in Veal Calves. Animals (Basel) 2023;13:3464.

27. Sáadatnia A, Mohammadi GR, Azizzadeh M, et al. Effect of ultrasonographic lung consolidation on health and growth in dairy calves: A longitudinal study. J Dairy Sci 2023;106:8047-8059.

28. Fiore E, Lisuzzo A, Beltrame A, et al. Lung Ultrasonography and Clinical Follow-Up Evaluations in Fattening Bulls Affected by Bovine Respiratory Disease (BRD) during the Restocking Period and after Tulathromycin and Ketoprofen Treatment. Animals (Basel) (Basel) 2022;12:994.

29. Elder LA, Hinnant HR, Mandella CM, et al. Differential gene expression in peripheral leukocytes of pre-weaned Holstein heifer calves with respiratory disease. Plos One 2023;18:e0285876.

30. Jourquin S, Lowie T, Bokma J, et al. Accuracy and inter‐rater agreement among practitioners using quick thoracic ultrasonography to diagnose calf pneumonia. Vet Rec 2024;194:e3896.

31. Lindley G, Booth R, Wathes C, et al. 56. Inter-rater agreement of different thoracic ultrasonography techniques performed by novice operators on preweaned dairy calves. Animal-science proceedings 2024;15:62-63.

32. Dunn T, Ollivett T, Renaud D, et al. The effect of lung consolidation, as determined by ultrasonography, on first-lactation milk production in Holstein dairy calves. J Dairy Sci 2018;101:5404-5410.

33. Flöck M. Diagnostic ultrasonography in cattle with thoracic disease. Vet J 2004;167:272-280.

34. Ollivett TL, Buczinski S. On-farm use of ultrasonography for bovine respiratory disease. Vet Clin North Am Food Anim Pract 2016;32:19-35.

35. Ollivett T. Thoracic ultrasound to monitor lung health and assist decision making in preweaned dairy calves. In: American Association of Bovine Practitioners Proceedings of the Fifty-First Annual Conference, Phoenix, Arizona 2018;185-187.

36. Cuevas-Gómez I, McGee M, Sánchez JM, et al. Association between clinical respiratory signs, lung lesions detected by thoracic ultrasonography and growth performance in pre‐weaned dairy calves. Ir Vet J 2021;74:7.

37. Pardon B, Jourquin S, Lowie T, et al. Lessons learned from 1 year of quick thoracic ultrasonography (qTUS) training for practitioners to improve pneumonia diagnosis in calves. In: World Buiatric Congress 2022 2022.

38. Jourquin S, Lowie T, Debruyne F, et al. Effect of on-arrival BRD vaccination on ultrasound confirmed pneumonia and production parameters in male dairy calves: a randomized clinical trial. J Dairy Sci 2023;106:9260-9275.

39. Babkine M, Blond L. Ultrasonography of the bovine respiratory system and its practical application. Vet Clin North Am Food Anim Pract 2009;25:633-649.

40. Anteveli G, Andrade J, Alves B, et al. Choosing the optimal combination of lungs lobe evaluation during focused pulmonary ultrasonography in calves. J Dairy Sci 2024.

41. Lisuzzo A, Achard D, Valenza A, et al. Bovine Respiratory Disease in Veal Calves: Benefits Associated with Its Early Detection by Lung Ultrasonography and Its Prompt Treatment with a Single Dose of a Fixed Combination of Florfenicol and Meloxicam. Animals 2024;14:3499.

42. Hoffelner J, Peinhopf-Petz W, Wittek T. Associations between Ultrasonographically Diagnosed Lung Lesions, Clinical Parameters and Treatment Frequency in Veal Calves in an Austrian Fattening Farm. Animals 2024;14:2311.

43. Rouault M, Foucras G, Meurens F, et al. Thoracic Ultrasonography Findings and Their Association With Respiratory Pathogens in 221 Young Beef Cattle at Fattening Farms: A Cross‐Sectional Study. Journal of Veterinary Internal Medicine 2025;39:e70141.

44. Feitoza LF, White BJ, Larson RL, et al. Targeted thoracic ultrasonography shows high diagnostic potential for interstitial pneumonia in feedyard cattle. American Journal of Veterinary Research 2024;1:1-9.

45. Feitoza LF, White BJ, Larson RL, et al. Associations Between Thoracic Ultrasound Chute-Side Evaluations and 60-Day Outcomes in Feedyard Cattle at Time of First Treatment for Respiratory Disease. Veterinary Sciences 2025;12:369.

46. Clinquart J, Lowie T, Jourquin S, et al. Association of Lung Consolidation Depth With Pathogens Isolated From Bronchoalveolar Lavage Fluid in Calves With Clinical Signs of Respiratory Disease. Journal of Veterinary Internal Medicine 2025;39:e70120.

47. Boccardo A, Ossola M, Pavesi LF, et al. An on-farm observational study on the prevalence and associated factors of bacteremia in preweaned dairy calves diagnosed with bronchopneumonia by thoracic ultrasonography. BMC Veterinary Research 2025;21:258.
